# LTP-like plasticity in the visual system and in the motor system appear related in young and healthy subjects

**DOI:** 10.3389/fnhum.2015.00506

**Published:** 2015-09-24

**Authors:** Stefan Klöppel, Eliza Lauer, Jessica Peter, Lora Minkova, Christoph Nissen, Claus Normann, Janine Reis, Florian Mainberger, Michael Bach, Jacob Lahr

**Affiliations:** ^1^Center of Geriatrics and Gerontology Freiburg, University Medical Center FreiburgFreiburg, Germany; ^2^Department of Neurology, Freiburg Brain Imaging, University Medical Center FreiburgFreiburg, Germany; ^3^Department of Psychiatry and Psychotherapy, University Medical Center FreiburgFreiburg, Germany; ^4^Department of Neurology, University Medical Center FreiburgFreiburg, Germany; ^5^Laboratory for Biological and Personality Psychology, Department of Psychology, University of FreiburgFreiburg, Germany; ^6^Department of Pediatrics, Kinderzentrum München gGmbH, Technical University MunichMunich, Germany; ^7^Eye Center, University Medical Center FreiburgFreiburg, Germany

**Keywords:** long-term potentiation (LTP), LTP-like plasticity, VEP potentiation, paired associative stimulation (PAS), motor learning, verbal learning, neuroplasticity

## Abstract

LTP-like plasticity measured by visual evoked potentials (VEP) can be induced in the intact human brain by presenting checkerboard reversals. Also associated with LTP-like plasticity, around two third of participants respond to transcranial magnetic stimulation (TMS) with a paired-associate stimulation (PAS) protocol with a potentiation of their motor evoked potentials. LTP-like processes are also required for verbal and motor learning tasks. We compared effect sizes, responder rates and intercorrelations as well as the potential influence of attention between these four assessments in a group of 37 young and healthy volunteers. We observed a potentiation effect of the N75 and P100 VEP component which positively correlated with plasticity induced by PAS. Subjects with a better subjective alertness were more likely to show PAS and VEP potentiation. No correlation was found between the other assessments. Effect sizes and responder rates of VEP potentiation were higher compared to PAS. Our results indicate a high variability of LTP-like effects and no evidence for a system-specific nature. As a consequence, studies wishing to assess individual levels of LTP-like plasticity should employ a combination of multiple assessments.

## Introduction

Studies, primarily in animals, indicate that synaptic long-term potentiation (LTP) is an important physiological mechanism underlying synaptic plasticity. LTP can be induced by tetanic stimulation at high frequencies or by associative pre- and post-synaptic stimulation (Cooke and Bliss, 2006). LTP has been studied extensively at the cellular and molecular level in animals and humans, mainly in the hippocampus (Bliss and Lomo, 1973; Beck et al., 2000), the visual (Komatsu et al., 1981), and somatosensory cortex (Fox, 2002), as well as temporal lobe (Chen et al., 1996), and spinal cord (Ji et al., 2003). Studies (Hess and Donoghue, 1994; Rioult-Pedotti et al., 2000) indicate that LTP is an omnipresent mechanism of plasticity in the CNS.

As a measure of LTP-like plasticity in the visual cortex, the increase of the amplitude of the P100 component of the visually-evoked potential (VEP) has been used in several studies (Normann et al., 2007; Elvsåshagen et al., 2012). Protocols typically show checkerboards with alternating patterns (i.e., checkerboard reversals) and use a two electrodes EEG montage as the readout. Slight variations of the presentation parameter such as reversal frequency may interfere the induction of LTP-like effects (Lahr et al., 2014).

Paired-associative stimulation (PAS) (Stefan et al., 2000, 2002; Sale et al., 2007; Müller-Dahlhaus et al., 2008; Player et al., 2012) is one of the most frequently employed paradigms to assess LTP-like plasticity in the motor cortex. Compared with VEP potentiation, PAS requires a more extensive setup as it comprises the combination of peripheral nerve stimulation, transcranial magnetic stimulation (TMS), and possibly an optical navigation system. A response to PAS is characterized by an increased motor-evoked potential (MEP) following the PAS-intervention. “LTP-like” indicates that these mechanisms share some (such as e.g., stimulus specificity for VEP-potentiation, McNair et al., 2006; Ross et al., 2008 or NMDA-receptor dependence for PAS, Stefan et al., 2002), but not all characteristics with LTP, as measured at the cellular level (Beste et al., 2011; Clapp et al., 2012).

The role of LTP in cognitive learning tasks has been studied most intensively using motor skill learning where changes in synaptic density can be observed within minutes and are likely related to LTP (Rioult-Pedotti et al., 2000; Reis et al., 2009; Xu et al., 2009; Cantarero et al., 2013a). Verbal learning (Rowland et al., 2005) shares at least some characteristics with LTP-like effects while the delayed recall of learned words or working memory performance (Rowland et al., 2005) have not.

Although LTP-like effects have been observed in several neuronal systems in humans, it remains unclear if the individual level of potentiation effects is associated between systems. Although paradigms for the induction of LTP-like effects are heterogeneous in respect to stimulus type and frequency, they may all involve LTP as observed at the cellular level as a common pathway. One study found a significant correlation between PAS and motor skill learning across patients and controls (Frantseva et al., 2008). In contrast, PAS did not correlate with the performance in a rotor pursuit motor learning task (Player et al., 2012). Likewise, López-Alonso et al. (2014) analyzed PAS, theta burst TMS and anodal tDCS of the primary motor cortex and concluded that the response to one assessment did not predict the response to another. Similarly, a study in healthy elderly did not find a significant correlation between PAS induced potentiation and verbal learning (List et al., 2013).

Several factors such as age (Müller-Dahlhaus et al., 2008), time of the day (Sale et al., 2007), and attention (Stefan et al., 2004) have been proposed to explain a high inter-individual variability of LTP-like effects. Also genetic factors play a role in LTP-like plasticity, especially the Val66Met polymorphism in the gene coding for brain derived neurotrophic factor (BDNF) is in focus, as it has been shown to modulate LTP-like plasticity (Ho et al., 2006; Kleim et al., 2006; Cheeran et al., 2008; Fritsch et al., 2010; Cirillo et al., 2012). Typically, the presence of more Val alleles was associated with higher plasticity. However, other studies (Li Voti et al., 2011; Nakamura et al., 2011) found no effect, or even increased plasticity (Antal et al., 2010; Teo et al., 2014) for carriers of the met-allele.

In this study, we compared VEP potentiation as an example of exposure based learning (Beste and Dinse, 2013) to a broad range of assessments of plasticity including PAS as well as motor and verbal learning tasks in a group of young healthy volunteers. Assessments were chosen to probe different neuronal systems outside the visual system (i.e., motor system, hippocampus) to identify global and system-specific elements of LTP-like effects and were all applied to each individual. Each assessment led to one variable coding system-specific plasticity. We expected positive correlations between assessments within (i.e., motor learning and PAS in the motor system) and across systems. We compared effect sizes and responder-rates.

## Materials and methods

### Subjects

Thirty-seven healthy right-handed volunteers [mean age 23.8 ± 0.3 years (SEM); 19 female] were included. A verbal IQ of 111.5 (± 1.5; range 97–129) indicated a high educational background. Subjects were recruited through advertisements and a subsequent telephone screening. Exclusion criteria included a history of neurological or psychiatric disorders, pregnancy, metal implants, consumption of CNS-acting medication or drugs, or the intake of alcohol within 24 h of the assessment. The study was approved by the ethics commission of the University Medical Center Freiburg (approval #227/12) and all participants gave their written informed consent.

Participants attended a single visit in the afternoon, which included an assessment of current subjective and objective alertness (Sturm and Willmes, 2001). For intrinsic alertness, the participants were instructed to press a button in response to a cross appearing on the screen. The same setting was used to measure phasic alertness but with an acoustic signal to alert attention just before the cross appeared. Subjective alertness was assessed using a 6-point Likert scale. Participants started with the verbal learning and memory test (VLMT; Helmstaedter et al., 2001), followed either by TMS or the motor learning task, all detailed below. Ordering of TMS and motor learning was randomized as both tasks involve the motor system and motor skill learning may temporarily occlude the induction of LTP-like plasticity (Ziemann et al., 2004; Stefan et al., 2006; Rosenkranz et al., 2007; Cantarero et al., 2013b). TMS or motor learning was then followed by the VEP potentiation paradigm and the other assessment of the motor system. To assess system-specific plasticity, we chose two assessments of the motor system given that motor performance is a particularly accessible marker.

### Verbal learning task

Subjects performed the VLMT shortened to three series of word presentations to avoid ceiling effects in this young cohort (Van der Elst et al., 2005). The test instructions were read aloud by the experimenter. The participants were required to listen to 15 words (one noun per second) and to repeat as many words as possible in any order directly after each round of presentations. For statistical analysis we used the sum of words learned in the three sessions.

### Paired associative stimulation

TMS was performed using a magnetic stimulator (Magstim 200; Magstim Whitland, UK) with a figure-of-eight coil. The coil was positioned tangentially above the left motor cortex, lateral to the vertex in a 45° angle with the handle pointing backwards. Next, the “hot-spot” of the abductor pollicis brevis (APB) was identified as the position where small MEPs could be recorded consistently from the APB with moderately supra-threshold stimulator intensity. Then, the stimulator intensity was adjusted to achieve an MEP amplitude of 1 mV. Next, this position was registered as a home position in an optical navigation system (Localite GmbH, Sankt Augustin, Germany). Throughout the experiment, the coil position was held within 5 mm and 5° of the home position relative to the skull, thereby also controlling for slow movements of the subjects. Electrical stimulation was performed using a Digitimer DS7 constant current stimulator (Digitimer, Welwyn Garden City, UK). The stimulator (cathode proximal) was attached to the inner side of the subject's right wrist at a position optimally stimulating the median nerve. Stimulation intensity was set to 300% of the individual perception threshold (Stefan et al., 2000).

Subjects were seated with their right forearm comfortably placed in neutral semi-pronated posture on a pillow. The MEP from the APB were monitored online and amplified, bandpass-filtered (lowpass-filter: 8 kHz; time-constant: 30 ms, corresponding to a cut-off frequency of 5.3 Hz) and digitized with an analog-digital converter at a sampling rate of 2 kHz (micro1401, Cambridge Electronic Designs, UK). The excitatory PAS25 protocol was divided in three different conditions: one pre-measurement as baseline (PRE), the intervention condition (PAS), and three POST-measurement conditions after 1, 8, and 15 min, respectively. During the PRE- and POST-conditions, the 20 TMS pulses were applied at an interval of 6 s and with a variability of 20% in order to prevent systematic MEP variability due to expectation. For potentiation, 180 paired stimuli were applied at an interval of 5 s and with an interstimulus interval of 25 ms between electrical and magnetic stimulus (Stefan et al., 2000). To keep subjects attentive, they were presented landscape images on a screen during the PRE and POST conditions. When the PAS intervention started, subjects were asked to mentally count blue balls appearing on a computer screen.

### Analysis of TMS data

Trials with pre-facilitated activity were rejected manually, affecting on average 6.3 ± 1.8 out of 80 trials per subject. We determined the mean peak-to-peak MEP amplitude within 100 ms of the magnetic stimulus. To compute a marker of potentiation for further analyses, the three post-sessions were averaged and divided by the baseline amplitude (Müller-Dahlhaus et al., 2008; List et al., 2013). A value above 1 indicated a PAS responder (Müller-Dahlhaus et al., 2008). Of note, the physiological basis for a distinction between PAS-responders and non-responders remains unclear but is reported here for consistency with existing studies.

### Visual potentiation

Potentiation of VEPs was induced by a checkerboard-reversal stimulation based on previous work (Normann et al., 2007). Before the VEP task, subjects were tested for visual acuity by presentation of Landolt rings using a computerized test battery (Freiburg Visual Acuity Test; FrACT; Bach, 1996) assuring a visual acuity above 0.6.

**Figure 1:**
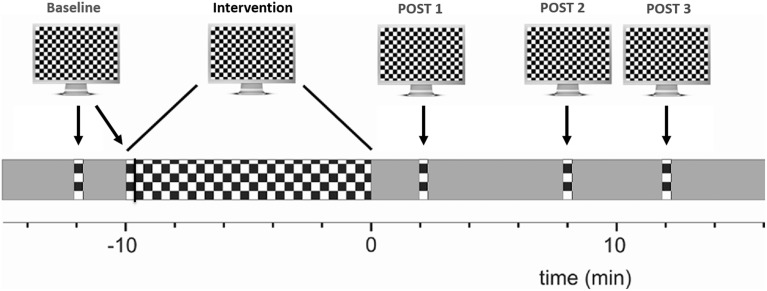
**Experimental overview of VEP potentiation: checkerboard pattern reversals were used to induce LTP-like plasticity**. VEP amplitude at baseline was compared to the averaged post intervention amplitude. See main text for details.

EEG was recorded from the occiput (Oz, standard 10–20 positioning system), with a reference electrode placed on the forehead (FPz). A ground electrode was placed on the left wrist. Signals were amplified and captured at a sampling rate of 1 kHz. Stimuli were delivered using Cogent Graphics software (http://www.vislab.ucl.ac.uk/cogent.php) on a CRT screen with a size of 36 × 27 cm, a resolution of 800 × 600 pixels and a frame rate of 75 Hz. The monitor was 114 cm away and its mean luminance was set to 45 cd/m^2^. Subjects were requested to fixate a cross at the center of the screen and to read out numbers appearing there at random intervals for a duration of 300 ms, an interval too short to read the number should saccadic correction be necessary. Of note, the presentation of numbers was not correlated with the presentation of checkerboard reversals and occurred mainly during the 10 min intervention period. The checkerboard size was 17.5° × 13.3° and the size of each check was 0.5°. It alternated at two reversals per second (rps). A 20 s period consisting of 40 sweeps was recorded per condition. For better data stability, two baseline measurements of checkerboard reversal VEPs (20 s each) were recorded at baseline (PRE). Subsequently, checkerboard reversals with the same reversal rate were shown continuously in a 10 min intervention phase, followed by three further intervals of 20 s recorded 2, 8, and 12 min after the intervention phase (POST1-3; Figure 1).

### Analysis of VEP data

Data were digitally bandpass-filtered from 1 to 30 Hz. Each sweep was separately baseline corrected (baseline: reversal instant ± 25 ms), and sweeps exceeding ± 80 μV were rejected and regarded as blink artifacts. The P100 peak was determined from the grand average data of each subject (maximum between 90 and 110 ms), as was the N75 peak (minimum between 60 and 100 ms). The peak-to-peak amplitude between N75 and P100 was normalized to 1 in the grand average. As the P100 amplitude had shown a significant potentiation effect in two previous studies (Normann et al., 2007; Elvsåshagen et al., 2012), we used the difference between the P100 amplitude before (averaged across both baseline measurements in order to improve the signal-to-noise ratio) and after the intervention phase (averaged across all three time points) as a primary marker of potentiation. When the P100 amplitude was increased after the intervention phase, subjects were considered as *VEP responders*.

### Motor learning

**Figure 2:**
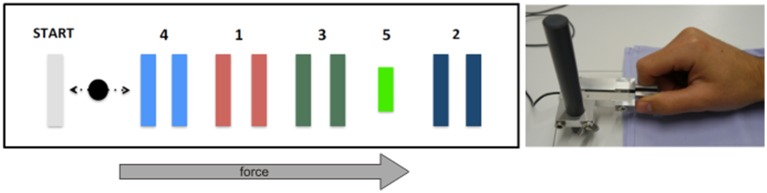
**Experimental overview of sequential visual isometric pinch task**. Subjects were required to move the black cursor horizontally toward the numbered gates **(left)** by modulating pinch on a force transducer between thumb and index finger **(right)**. See main text for details.

We adopted a sequential visual isometric pinch task (SVIPT) previously used to assess LTP-like effects involved in motor skill learning (Reis et al., 2009; Cantarero et al., 2013a,b). Subjects were seated in front of a computer screen holding a force transducer with their right thumb and index finger (Figure 2). The force transducer controlled a cursor, which moved horizontally to the right when increasing force and returned to a home position when released. Subjects were asked to navigate the cursor as quickly and accurately as possible from home position to each of five different target positions subsequently. Target errors resulted when applied force was too weak or too strong to hit the target. Each subject performed four blocks of 21 trials (first trial excluded from analyses) with five targets in each trial. We computed a skill measure to quantify motor learning as in previous work (Reis et al., 2009). The respective equation (see Equation 1) combines the total number of target errors and the duration of each training block. The exponent was set to 3.43 as determined experimentally in an unrelated group of healthy volunteers (Schöchlin-Marx, 2014). Similar to the analyses of VEP and PAS data, we used the improvement of the skill between first and fourth block as marker of plasticity. To this end, we normalized the skill parameter by dividing the last block by the first one.

(1) $$\begin{array}{r} skill=\frac{1\;-\;errorrate}{errorrate\left({ln\left(movementtime\right)}^{3.43}\right)} \end{array}$$

### Statistical procedures

As markers of plasticity were not normally distributed according to Shapiro-Wilk testing, non-parametric tests were used for statistical analyses (Kendall's tau for correlations, Wilcoxon's signed ranks test for pairwise comparisons, and Mann-Whitney-U-test for unpaired comparisons). Using Kendall's τ, non-parametric correlations were calculated pairwise between all four assessments represented by the summated learned words, the improvement of motor skill and the potentiation induced by TMS and VEP. Effect sizes were computed for all assessments as *r* = Z/√n and interpreted according to Cohen (1988). Absolute values are reported with one SEM. Motivated by previous work (López-Alonso et al., 2014), we also tested if subjects classified as TMS responders would yield an increased probability of showing VEP potentiation and vice versa using Cohen's kappa (Fleiss et al., 2003).

## Results

PAS data from four and VEP data from four participants were unusable due to movement artifacts or equipment failure, and one subject perceived TMS stimuli as too unpleasant to complete the protocol. All participants rated themselves as highly alert on the subjective alertness scale (4.3 ± 0.2, where 6 is the highest score). Likewise, the participants were objectively alert on both intrinsic alertness (reaction time 204 ± 3 ms; percentile rank 72.0 ± 3.2) and phasic alertness (reaction time 190.0 ± 6.3 ms, percentile rank 57.5 ± 4.7).

**Figure 3:**
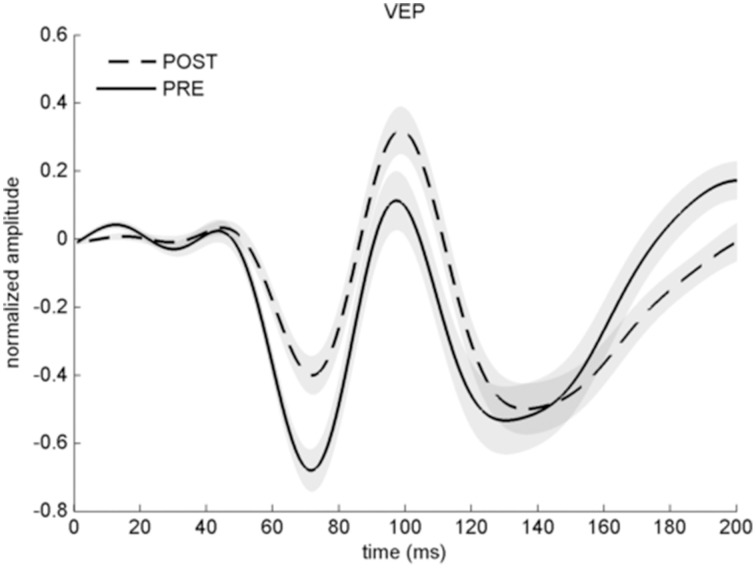
**Averaged VEP before (solid line) and after stimulation with checkerboard reversals (dashed line) across all participants**. Note that the y-axis represents arbitrary units as VEP amplitude is normalized. Error shades indicate ±1 SEM.

**Figure 4:**
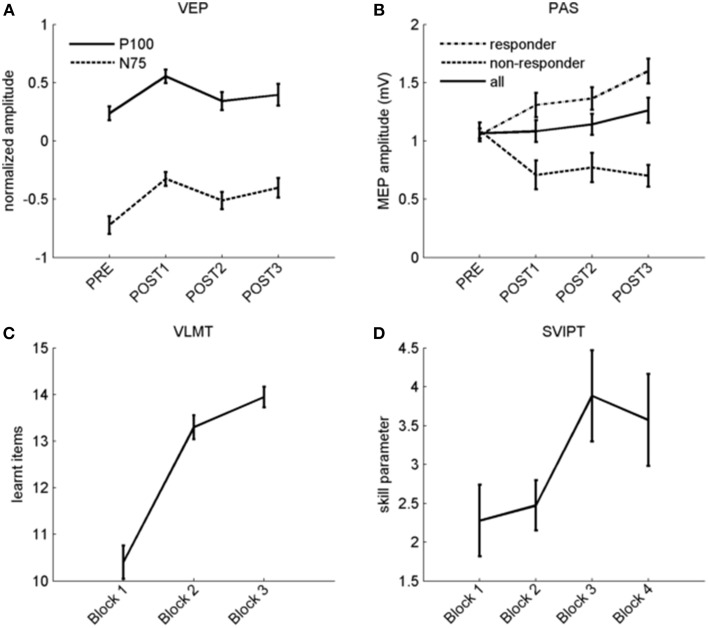
**Overview of plasticity protocols. (A)** Normalized P100 (solid line) and N75 (dashed line) amplitudes before and after stimulation with checkerboard reversals. **(B)** MEP amplitude before and after PAS across all subjects (solid line), and separated in responders (dashed line) and non-responders (dotted line). **(C)** Verbal learning curve with three repetitions (x-axis). Number of learned items is shown on the y-axis. **(D)** Skill improvement during four blocks of motor learning, the slight drop in the fourth block is due to an increase of the error rate. For the whole figure, error bars indicate ±1 SEM.

VEP analyses indicated a less negative N75 and more positive P100 after potentiation, in line with previous work (Normann et al., 2007) (baseline 0.24 ± 0.06, after potentiation: 0.43 ± 0.06; arbitrary units). 25 out of 33 individuals (75%) had a P100 averaged across all post intervals above the level before potentiation and were therefore considered VEP responders. Computed effect size across all participants was large (*r* = 0.61; *p* < 0.001), see Figures 3, 4A. A Met allele was significantly associated with stronger VEP potentiation effects (mean potentiation effect met allele present: 0.3 ± 0.07; absent: 0.12 ± 0.06).

In the VLMT, remembered items increased from 10.4 ± 0.4 in the first to 13.9 ± 0.2 items after the third presentation [*r* = 0.85 (large effect); *p* < 0.001; Figure 4C].

Figure 4B displays raw data before and after PAS. 20 subjects (63%) were PAS responders while 12 did not show higher MEP after the potentiation phase. The potentiation effect as indicated by an increase in MEP amplitude between PAS and the averaged post-intervals was not significant, and the effect size across all participants was small (*r* = 0.22; *p* = 0.22; MEP-pre: 1.07 ± 0.04 mV; MEP mean post: 1.16 ± 0.09 mV).

As expected (Reis et al., 2009), we observed faster and more accurate movements over time although motor skill dropped again in the last block, possibly due to fatigue (see Figure 4D; skill parameter block 1: 2.28 ± 0.46; block 4: 3.57 ± 0.59). Observed skill improvement between first and fourth block [*r* = 0.41 (medium effect); *p* = 0.012] was largely due to shorter movement times rather than a reduction of errors (data not shown). Groups differing in genotype did not show differences in the strength of another measure of plasticity except VEP potentiation.

Subjects with higher subjective alertness were more likely to be PAS (τ = 0.36, *p* = 0.03) and VEP-responders (τ = 0.35, *p* = 0.03). Cohen's kappa between PAS responders and VEP responders was 0.037, with values < 0.4 considered as a poor agreement between the measures (Fleiss et al., 2003). We observed no correlation between motor learning improvement and PAS, while PAS correlated (τ = 0.27, *p* = 0.048) with P100 VEP potentiation (Table 1). No similar correlation was observed for the N75 amplitude change. An effect of task-order was observed only for the motor learning task in which subjects became more skillful when motor learning was performed before PAS (*p* = 0.02). All other markers of plasticity—notably PAS—did not show a task-order effect. Scatterplots with all pairwise correlations are reported in a supplement (Supplementary Material S1).

**Table 1:** **Correlation matrix (Kendall's τ) between markers of plasticity**.

		**PAS**	**VEP_P100_**	**SVIPT**	**VLMT**
PAS	τ				
	*p*				
	*n*	32			
VEP_*P*100_	τ	**0.265**			
	*p*	**0.048**			
	*n*	**28**	33		
SVIPT	T	0.016	0.110		
	*p*	0.897	0.369		
	*n*	32	33	37	
VLMT	τ	−0.146	−0.025	0.119	
	*p*	0.253	0.839	0.311	
	*n*	32	33	37	37

Bold letters indicate significant effects (p < 0.05).

## Discussion

In this study, we applied a range of assessments of LTP-like plasticity to the same individuals.

Results of each of the four assessments separately replicate existing work. This includes verbal and motor learning performance (Helmstaedter et al., 2001; Schöchlin-Marx, 2014), a PAS- TMS responder rate of 63% (Müller-Dahlhaus et al., 2008), and a robust VEP potentiation (Normann et al., 2007). Subjects with a higher subjective score of alertness were more likely to be VEP or PAS responders, underlining the association of PAS-response with alertness (Mainberger et al., 2013) and focused attention (Stefan et al., 2004), however, there was no such association with phasic or intrinsic alertness. The increase of the motor skill was associated with task-order, with subjects performing the motor task earlier achieving better results which is most likely due to fatigue. 76% of the subjects responded to VEP potentiation, leading to higher effect sizes of VEP compared to MEP potentiation across all participants. Further, we observed a positive correlation between both potentiations. This may implicate an underlying common pathway of LTP-like plasticity across the motor and visual systems. However, response to VEP potentiation and PAS showed poor agreement, and the correlation between PAS and VEP potentiation was scarcely significant, and would not survive correction for multiple testing. In contrast, we found no correlation between the two assessments within the motor system. The absence of correlation between PAS and motor skill learning contradicts an earlier study (Frantseva et al., 2008) which found such an association when pooling across individuals with and without schizophrenia. As no correlation within groups is reported, their findings could primarily be driven by between-group difference. Our results argue against a system-specific expression of individual LTP-like levels. However, motor skill learning relies more heavily on higher cognitive functions compared to TMS. In fact, PAS-TMS and VEP potentiation are the two assessments which are relatively less dependent on higher cognitive function and attention and this could make it easier to detect a correlation between them. High variability (Müller-Dahlhaus et al., 2008) and relatively low test-retest reliability (Fratello et al., 2006) have been found for PAS but stability parameters are no worse compared to alternative protocols (Player et al., 2012; López-Alonso et al., 2014). While reliability of VLMT is high (Helmstaedter et al., 2001), similarly detailed evaluations of VEP potentiation and for our specific version of motor learning are still missing but findings were at least reproduced in subsequent studies (Schambra et al., 2011; Elvsåshagen et al., 2012). In addition to the variability of each assessment, the definition of indices of plasticity from each assessment will also influence the results. For TMS, a multiplicative model is established to measure plasticity which we adopted for motor skill learning. For word learning, a normalization to baseline performance did not seem appropriate, as the performance in the first block already requires learning. We therefore summed the number of learned items, thereby adopting the approach by Chen et al. (1996). For VEP potentiation, we chose the amplitude increase of P100 as marker of plasticity as previous studies have shown significant effects for P100 modulation (Normann et al., 2007; Elvsåshagen et al., 2012). Of note, the shift of the N75 component may be a more parsimonious measure of LTP-like plasticity with fewer involved synapses in the generation of the earlier components of the VEP. As shown in Figure 4A, both components performed very similar in our study. Another study reporting results from VEP potentiation (Teyler et al., 2005) applied an independent component analyses which makes comparisons difficult.

Although we found a correlation between VEP and MEP potentiation, the agreement between these assessments was poor, and in combination with other studies (Player et al., 2012; List et al., 2013; López-Alonso et al., 2014), our results confirm that the level of LTP-like plasticity in an individual differs within and across systems.

As a consequence, studies wishing to assess individual levels of LTP-like plasticity should employ a combination of multiple assessments. A range of studies identified disease specific changes in one specific assessment of LTP-like plasticity (Normann et al., 2007; Freitas et al., 2011; Cavuş et al., 2012; Elvsåshagen et al., 2012). Future work should aim to identify to what degree the observed changes can be reproduced across systems and across assessment methods.

## Funding

The article processing charge was funded by the open access publication fund of the Albert Ludwigs University Freiburg.

### Conflict of interest statement

The authors declare that the research was conducted in the absence of any commercial or financial relationships that could be construed as a potential conflict of interest.
