# Supplementary material for: LTP-like plasticity in the visual system and in the motor system appear related in young and healthy subjects
Source: Front Hum Neurosci. 2015 Sep 24;9:506. doi: 10.3389/fnhum.2015.00506 (PMC4585301; doi:10.3389/fnhum.2015.00506)
Supplement: Supplementary file 1 [file Image1.PDF]

Supplementary Figure

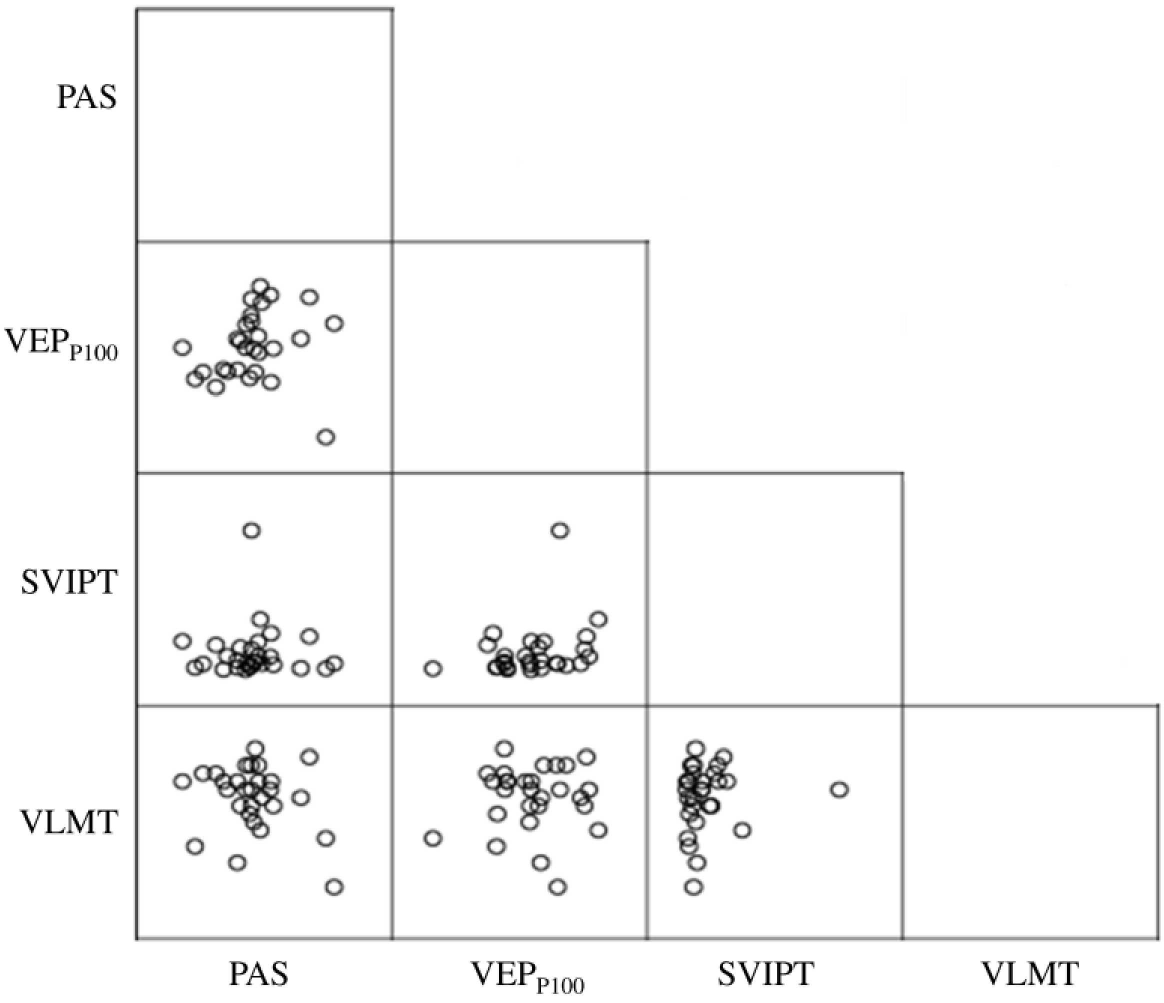

Figure S1: Scatterplots of pairwise correlations between markers of plasticity. *Note*: PAS = paired associative stimulation, VEP = visual evoked potentials, SVIPT = sequential visual isometric pinch task, VLMT = verbal learning and memory task.
